# Extra-abdominal Desmoid Tumor in a 23-Year-Old Male: A Case Report

**DOI:** 10.7759/cureus.109286

**Published:** 2026-05-20

**Authors:** Reagan Boyett, John Sisson, Tyler L Overbeek, Butler Wilbanks, Connor R Read

**Affiliations:** 1 Biomedical Sciences, Edward Via College of Osteopathic Medicine, Auburn, USA; 2 Orthopedics, Northeast Orthopedics, Gadsden, USA

**Keywords:** apc gene, ct-guided biopsy, ctnnb1, desmoid-type fibromatosis, familial adenomatous polyposis, gardner's syndrome, intramuscular lipoma, musculoskeletal oncology, soft tissue sarcoma, β catenin

## Abstract

Desmoid tumors are rare, locally aggressive neoplasms that can develop in various anatomical locations. Accurate diagnosis requires a high level of suspicion, as these tumors can resemble other soft tissue sarcomas. This case report discusses a 23-year-old male with a symptomatic thigh mass, highlighting the importance of biopsy and immunohistochemical staining to distinguish desmoid tumors from malignant tumors. We also examine genetic predispositions, evolving management strategies, and the potential of novel therapies such as nirogacestat. Due to the high recurrence rate and unpredictable nature of desmoid tumors, long-term monitoring and a multidisciplinary approach are essential for achieving optimal patient outcomes.

## Introduction

Desmoid-type fibromatosis, often called a desmoid tumor (DT), is a benign monoclonal proliferation of fibroblast tissue that is unable to metastasize despite being locally aggressive [1]. They are typically found in the abdomen when associated with familial adenomatous polyposis (FAP) and Gardner syndrome, and found in the extremities as a sporadic DT in patients testing negative for FAP [2-5]. DTs are rare neoplasms, having an estimated 2.2 to 5.36 cases per million person-years [6,7]. While the etiology of DT is not explicitly known, several genetic syndromes and trauma can predispose one to develop this tumor, though most are sporadic [8,9]. Most patients with DT (85%-90%) possess a somatic mutation in the CTNNB1 gene that encodes for the β-catenin protein and show subsequent deposition of the β-catenin protein in the nucleus [10,11].

Along with CTNNB1, a germline APC gene mutation and FAP have been linked to patients developing DT [11,12]. There is a higher prevalence of DT among female patients, though the disease is not exclusive to females [13]. Estrogen and oral contraceptive use are associated with advanced growth of DT, while spontaneous regression has been reported in patients undergoing menopause [3,4,14]. DT can cause various symptoms due to its invasion into surrounding tissue, or patients may report a painless mass [4]. Those with intra-abdominal DT may show signs of cachexia and weight loss, while those with DT in the extremities may show symptoms of pain, muscle weakness, and restricted range of motion [4,6]. Diagnosis of a DT is made via tissue biopsy. It is usually performed due to a wide range of clinical suspicions, from other fibroblastic tumors (i.e., fibroblastic sarcoma) to a scar tissue-related keloid mass [3]. While β-catenin deposition may be present on immunohistology of the mass, it is not specific enough to diagnose a DT. Therefore, genetic testing of CTNNB1 and APC must be conducted according to β-catenin status [3,6,10]. The presence of β-catenin directs testing toward the CTNNB1 gene, and the absence of β-catenin directs testing toward APC testing, as these genes are mutually exclusive [10]. Magnetic resonance imaging is the preferred imaging technique for extra-abdominal tumors, whereas computed tomography is the method of choice for intra-abdominal DT [6]. Classic imaging of a DT shows T2 hyperintense and hypo-isointense signal within an ill-defined tumor, seen in fibrous proliferation [15]. If asymptomatic, a period of active surveillance is advised, with recommendations of monitoring lasting between one and six months with decreasing frequency. Other treatment modalities are very dependent on the specifics of the tumor, so a multi-disciplinary approach with individualized care is needed. Different options include, but are not limited to, surgery, radiation, cryotherapy, and systemic therapies [6].

This work was previously presented as a poster presentation at the 2026 VCOM Research Recognition Day on February 6, 2026.

## Case presentation

A 23-year-old male presented to the orthopedic clinic for an evaluation of leg pain. The pain had been present for about two weeks. He described the pain as sharp and achy, rating it 2 out of 10 on the pain scale. He did not report any specific significant injury, but he noted that he could have slipped at work or in his garage. He had noticed a significant firm mass on the left thigh, which had not changed in shape or size since the symptoms began. He reported no chest pain or shortness of breath, as well as no recent weight loss. The patient had no past medical history or surgical history. His review of systems was positive only for calf cramps and muscle weakness. Pertinent family history included a bleeding disorder and cancer in his grandfather. On physical examination, he was well-appearing, alert, and in no acute distress. Musculoskeletal findings revealed mild tenderness to palpation over the groin and adductor muscle.

A firm, solid, and mobile mass was palpated within the adductor muscle, extending medially into the quadriceps and posteriorly into the gluteus maximus. There was no pain with gentle log roll or internal or external rotation. Strength was 5/5 in the hip flexors, hip abductors, and lower extremities throughout L4-S1. Sensation was intact at L4-S1. At this visit, a plain radiograph, seen in Figure 1, was indicated and performed. The anteroposterior view of the left femur showed no evidence of bony erosion or impending pathologic fracture. An urgent MRI of the left femur and a complete blood count, basic metabolic panel, C-reactive protein, and erythrocyte sedimentation rate were scheduled. The laboratory results were within normal limits.

**Figure 1:**
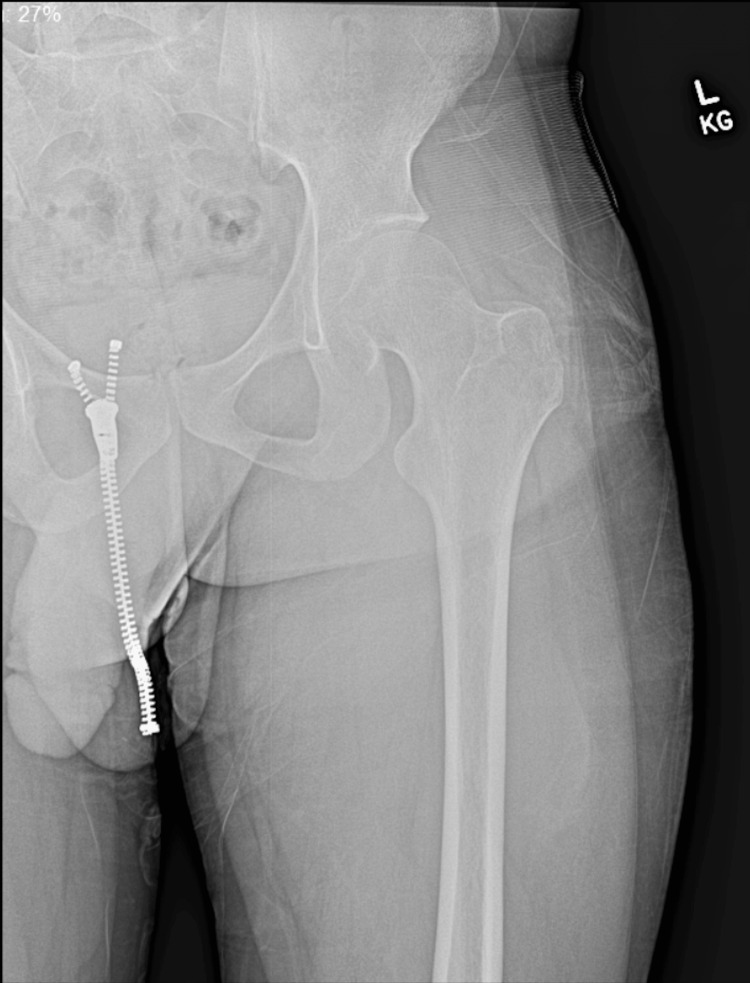
Plain radiograph of the left femur. Plain radiograph of the left femur was unrevealing of any calcifications, bony erosion, or impending pathologic fracture.

As seen in Figures 2-4, the urgent MRI did reveal a partially ill-defined mass, particularly along the interface with adjacent muscle fibers, where the mass appears to infiltrate along fascial planes and between muscle bundles within the anterior, medial, and posterior compartments of the proximal thigh musculature. The most anterior component of the mass measured 5.3 x 3.6 x 6.3 cm. The deeper component measured 7.0 x 6.0 x 13.6 cm. The posterior component measured 6.1 x 5.2 x 0.5 cm. The mass contacted the sciatic nerve and medial cortex of the femur, but radiology noted no marrow edema or acute fracture that would suggest osseous invasion.

**Figure 2:**
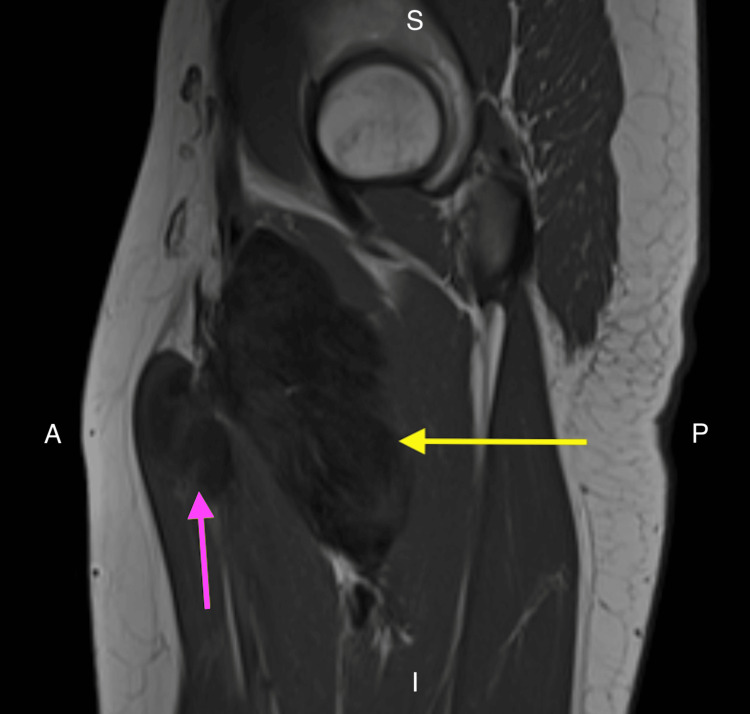
T1-weighted MRI (sagittal view) of the anterior component (pink arrow) and deep component (yellow arrow) of the desmoid tumor infiltrating the thigh. S: superior; I: inferior; A: anterior; P: posterior.

**Figure 3:**
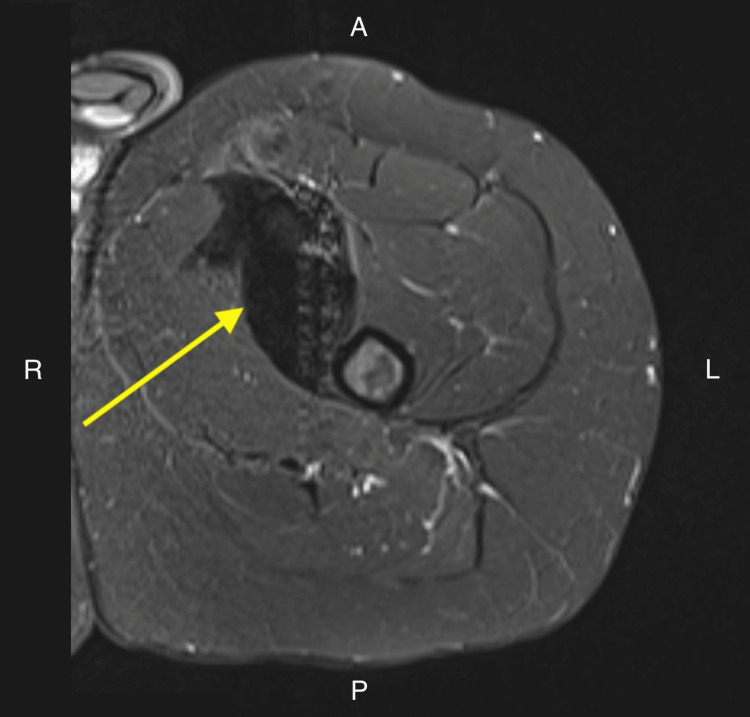
STIR MRI (axial view) of the deep component of the desmoid tumor infiltrating the thigh. STIR: short tau inversion recovery; A: anterior; P: posterior; R: right; L: left.

**Figure 4:**
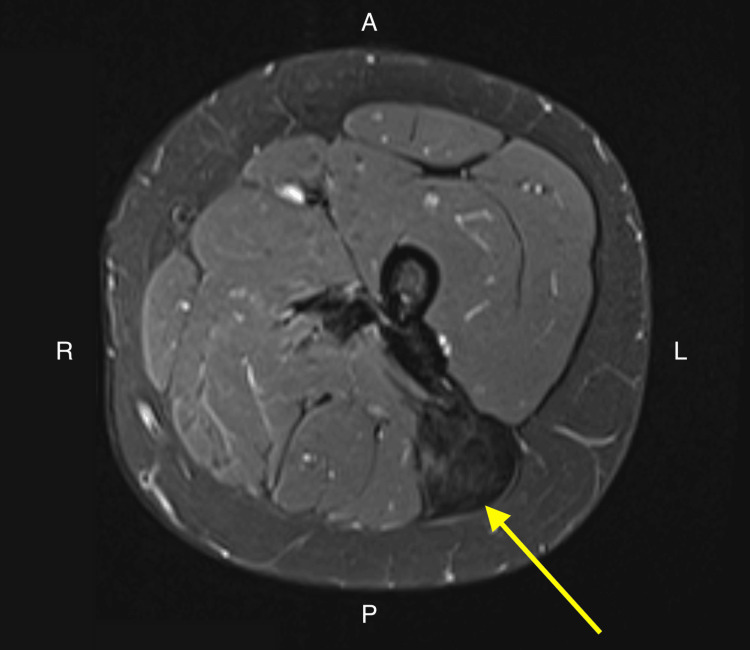
STIR MRI (axial view) of the posterior component of the desmoid tumor infiltrating the thigh. STIR: short tau inversion recovery; A: anterior; P: posterior; R: right; L: left.

T1-weighted imaging demonstrated predominantly intermediate signal intensity, isointense to slightly hyperintense relative to skeletal muscle. No areas of intrinsic T1 shortening (hemorrhage or fat) were identified. Interspersed linear bands of low T1 signal were present within the mass, consistent with dense collagenous stroma, which is a hallmark of desmoid-type fibromatosis. Short tau inversion recovery (STIR) imaging demonstrated heterogeneous intermediate-to-high signal intensity. The signal was not uniformly bright as seen in sarcomas, and there were interspersed areas of low signal intensity on all pulse sequences, corresponding to the characteristic "band sign." These are linear or curvilinear bands of T2 hypointensity traversing the lesion, which represent dense collagen bundles within the fibroblastic proliferation. The overall heterogeneous T2 signal with admixed low-signal bands was consistent with the MRI pattern of desmoid fibromatosis.

However, the radiologist noted shotty left inguinal lymph nodes measuring up to 2.2 x 1.3 cm. This was concerning for malignancy and metastatic disease. The size of the mass and rapid enlargement were concerning for sarcoma, so further evaluation was warranted.

The patient and his family were seen the next day in the clinic to discuss the MRI results. The most appropriate next step was to set him up for a CT-guided biopsy of the mass since it was greater than 5 cm. Two specimens were obtained; one came from the anterior compartment of the thigh and the other from the posterior. Hematoxylin and eosin stain histology revealed bland spindle cell proliferation with abundant collagen and minimal atypia, as seen in Figure 5.

**Figure 5:**
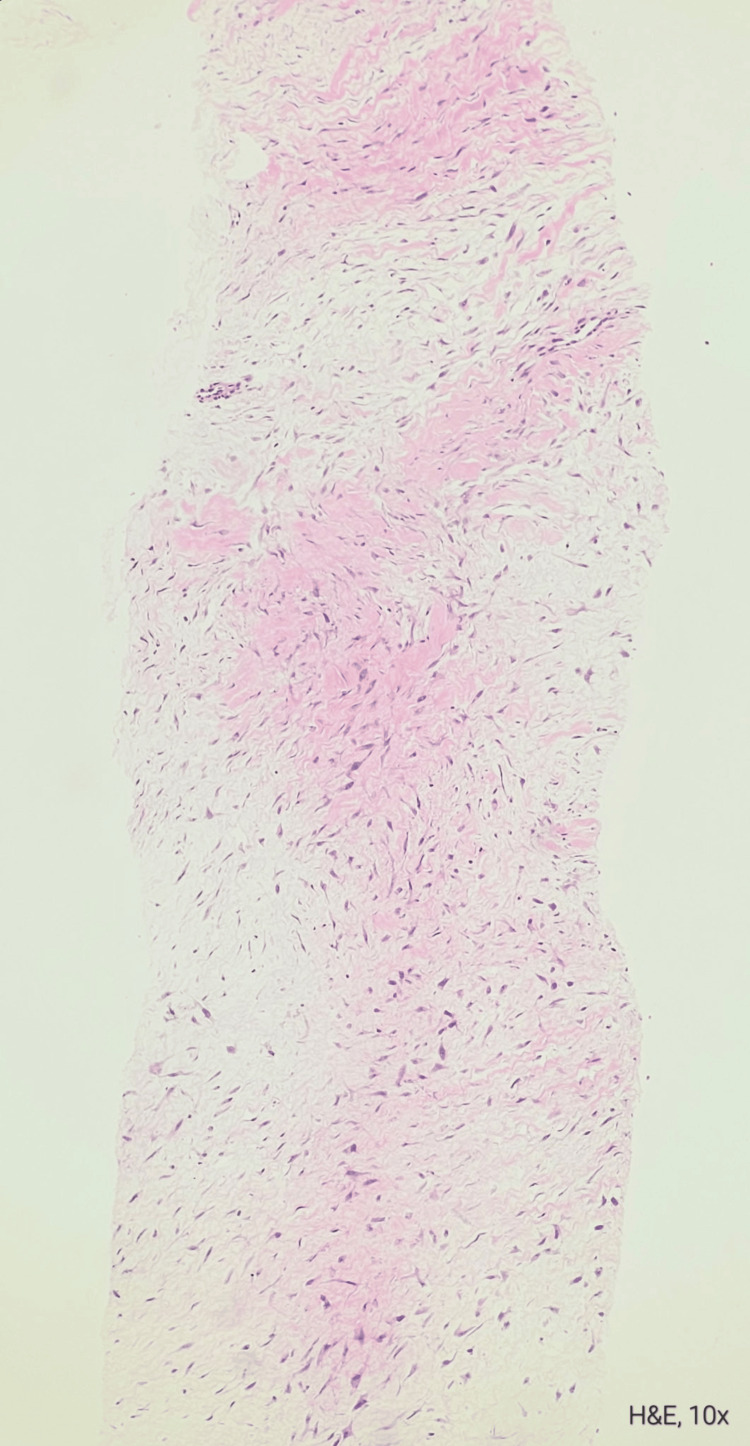
Hematoxylin and eosin stain of the biopsy at 10x magnification. Hematoxylin and eosin stain of the biopsy demonstrating soft tissue cores composed of bland spindle cells arranged in elongated fascicles with alternating areas of myxoid and collagenous stroma.

Muscle-specific actin had patchy areas of positive staining. Beta-catenin was diffusely positive and had extensive nuclear staining, as seen in Figure 6. Histology revealed soft tissue cores composed of bland spindle cells arranged in elongated fascicles with alternating areas of loose/myxoid and collagenous stroma. The cells were relatively monomorphic, showing no cytologic atypia. No mitoses or necrosis were seen. Calretinin also stained positive. These findings are consistent with fibromatosis, or desmoid tumor. The patient subsequently underwent genomic sequencing of the CT-guided biopsy, which revealed a CTNNB1 p.T41A mutation. The presence of beta-catenin and the CTNNB1 gene and the lack of APC gene mutation on genomic sequencing excluded the diagnosis of FAP.

**Figure 6:**
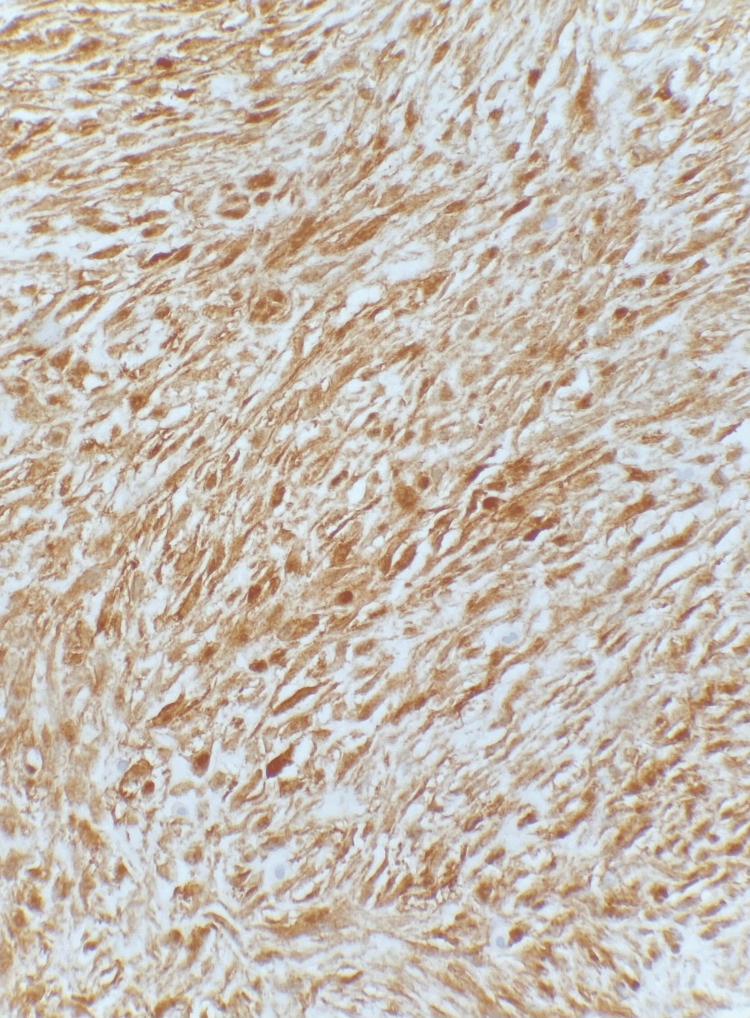
Beta-catenin stain of the biopsy at 40x magnification. Beta-catenin stain of the biopsy demonstrating diffuse positive beta-catenin staining with extensive nuclear staining.

The patient was referred to oncology. The oncologist elected to forgo surgical intervention and instead pursue medical management of the tumor with nirogacestat (Ogsiveo), a gamma secretase inhibitor. The patient tolerated the treatment well aside from the development of mouth sores and an episode of hypoglycemia, and these adverse effects were managed with oral dexamethasone and routine blood sugar monitoring, respectively, as well as lowering the dose of Ogsiveo. The oncologist also ordered an MRI of the abdomen and pelvis to screen for colon cancer, which came back negative for colonic abnormalities.

## Discussion

Our patient’s initial diagnosis of soft tissue sarcoma was challenged after the CT-guided biopsy was performed, which stained positively for markers associated with fibromatosis. This demonstrates the value of a CT-guided biopsy in any patient with findings consistent with malignancy. He had several alarming features related to the mass, including a size greater than 5 cm, no recent trauma, and a mobile mass. A hematoma was considered less likely due to the absence of significant trauma and bleeding disorder in this patient’s history. The final test to support the diagnosis of a DT is the gene mutation study of CTNNB1, specific but not sensitive to sporadic DTs [16]. Differentiation between DTs and other soft tissue sarcomas is important because DT does not spread to different parts of the body; it is only locally aggressive. DTs can also be a component of FAP caused by an APC gene mutation. Sporadic DTs are supported by CTNNB1 gene testing performed on tumor tissue. In contrast, DTs associated with FAP are supported by the identification of a germline APC gene mutation, which can be detected through blood or buccal swab testing [11].

There are different management options for this diagnosis. Watchful waiting is typically the first-line management option with serial MRI examinations. This is most appropriate for asymptomatic tumors showing no clinical signs of progression. DTs commonly regress, which is why watchful waiting may be the best course of action. In our patient with a rapidly expanding, painful tumor, systemic therapy is indicated with nirogacestat [17,18]. The Food and Drug Administration recently approved nirogacestat in the fall of 2023. This drug is a gamma-secretase inhibitor that cleaves multiple transmembrane proteins that are thought to play a role in the activating pathways that contribute to the growth of DTs, but the true mechanism is poorly understood [18,19]. The likelihood of being event-free at two years was 76% with nirogacestat and 44% with placebo according to a phase III trial published in the New England Journal of Medicine [18]. Postoperative management is highly dependent on the location of the tumor. Monitoring for recurrence is done by CT or MRI every three to six months for three years. After three years, the screening is recommended every six months to complete a total of five years, after which, screening is done annually [17]. Frequent surveillance is essential because recurrence occurs in 24-77% of cases [4]. Sporadic DTs occurring in the extremities have a higher rate of recurrence than intra-abdominal tumors [20].

## Conclusions

DTs are uncommon, but it is crucial to recognize them promptly and conduct biopsy and molecular testing to differentiate them from more aggressive soft tissue sarcomas or recognize them as part of FAP. Because they have a variable clinical course, treatment plans should be tailored to the individual patient. For asymptomatic cases, active surveillance may be considered, and systemic therapy should be reserved for progressive or symptomatic cases. The introduction of targeted therapies, such as gamma-secretase inhibitors like nirogacestat, represents a significant advancement in treatment options. This case underscores the value of early referral to a multidisciplinary team in managing DTs to optimize patient care and minimize morbidity.
